# Acute pancreatitis with organ dysfunction associates with abnormal blood lymphocyte signaling: controlled laboratory study

**DOI:** 10.1186/cc9329

**Published:** 2010-11-18

**Authors:** Jani Oiva, Harri Mustonen, Marja-Leena Kylänpää, Lea Kyhälä, Krista Kuuliala, Sanna Siitonen, Esko Kemppainen, Pauli Puolakkainen, Heikki Repo

**Affiliations:** 1 Department of Surgery, Helsinki University Central Hospital, PO Box 340, 00290 HUS, Helsinki, Finland; 2 Department of Bacteriology and Immunology, University of Helsinki, The Haartman Institute, PO Box 21, 00014 University of Helsinki, Helsinki, Finland; 3 Laboratory Services (HUSLAB), Helsinki University Central Hospital, PO Box 720, 00290 HUS, Helsinki, Finland

## Abstract

**Introduction:**

Severe acute pancreatitis is associated with systemic inflammation, compensatory immune suppression, secondary infections, vital organ dysfunction, and death.

Our study purpose was to delineate signaling profiles of circulating lymphocytes in acute pancreatitis complicated by organ dysfunction.

**Methods:**

Sixteen patients with acute pancreatitis, dysfunction of vital organ(s), and immune suppression (proportion of HLA-DR Human Leukocyte Antigen - DR - positive monocytes < 80%) participated. Healthy volunteers served as reference subjects. Using phospho-specific whole blood flow cytometry we studied lymphocyte phosphorylation of nuclear factor-κB (NFκB), mitogen-activated protein kinases p38 and extracellular signal-regulated kinases (ERK)1/2, and signal transducers and activators of transcription (STATs) 1, 3, and 6. Statistical comparisons were performed with the Wilcoxon-Mann-Whitney test.

**Results:**

In blood samples supplemented with tumor necrosis factor, *E. coli *or *S. aureus*, phosphorylation levels of NFκB were lower and levels of p38 were higher in patients with acute pancreatitis than healthy subjects. Low NFκB activation involved CD3+CD4+ and CD3+CD8+ lymphocytes. ERK1/2 phosphorylation induced by co-stimulation with phorbol 12-myristate 13-acetate and calcium ionophore A23187 was depressed in patients. STAT3 was constitutively activated in patients' CD3+CD4+ and CD3+CD8+ lymphocytes. Also, IL-6-induced STAT1 phosphorylation was impaired while IL-4-induced STAT6 phosphorylation was enhanced.

**Conclusions:**

Lymphocytes of patients with acute pancreatitis, organ dysfunction and immune suppression show impaired NFκB activation, which increases infection risk and enhanced p38 activation, which sustains inflammation. Secondly, they indicate constitutive STAT3 activation, which may favor Th17 lineage of CD4+ lymphocyte differentiation. Thirdly, they reveal impaired STAT1 activation and enhanced STAT6 activation, denoting a shift from Th1 towards Th2 differentiation.

## Introduction

Acute pancreatitis (AP) is usually a self-limiting disease resolving within days. Some patients, however, develop overwhelming systemic inflammation, which contributes to the development of vital organ dysfunction, the major cause of mortality in AP [1,2]. Systemic inflammation is designated by activation of circulating cells of both the innate immune system, such as monocytes [3], and the adaptive immune system, such as CD4+ T-helper (Th) -lymphocytes, CD8+ -lymphocytes [4,5], and CD19+ B -lymphocytes [6]. Cellular activation results in the systemic release of pro- and anti-inflammatory mediators, such as tumor necrosis factor (TNF) and interleukin (IL) -10 [7]. The latter promotes immune suppression, increasing the risk of secondary infections and multiple organ dysfunction syndrome [8-11]. Also, experimental [12] and clinical [13] studies suggest that the host's defense against infection is further depressed as the Th1 subpopulation of CD4+ T-cells becomes more strongly suppressed than the Th2 cells in the course of AP, leading to Th1/Th2 cytokine imbalance.

The molecular mechanisms involved in the pathogenesis of systemic inflammation and subsequent immune suppression include multiple signaling pathways and families of transcription factors, such as signal transducers and activators of transcription (STATs), nuclear factor-κB (NFκB), and members of the mitogen-activated protein (MAP)-kinase family [14]. In lymphocytes, STAT1 is activated by IL-6 and pro-inflammatory interferons [15,16], which support Th1 polarization of Th-lymphocytes, STAT3 by anti-inflammatory IL-10 [17] and by IL-6, which supports the Th17 lineage of lymphocyte differentiation [18] and STAT6 by IL-4, which supports Th2 polarization of Th-cells [19]. The lymphocyte NFκB is activated by TNF [20,21], while MAP-kinases ERK (extracellular signal-regulated kinase) 1/2 and p38 are phosphorylated by cytokine receptor activation [22], or by co-stimulation of lymphocytes with calcium ionophore and PMA (phorbol 12-myristate 13-acetate).

We recently described monocyte signaling profiles in 13 AP patients with vital organ dysfunction using phospho-specific whole blood flow cytometry [23]. In the present study we describe signaling profiles of the patients' circulating lymphocytes.

## Material and methods

### Patients and controls

The study comprises 16 men with AP admitted to the intensive care unit (ICU) at Helsinki University Central Hospital. The first 13 patients, whose monocyte signaling profiles are described in our previous study [23], were admitted between September 2007 and January 2009 and the last three patients between January and May 2010 (Table 1). In addition to severe AP, the inclusion criterion for the study was that the proportion of HLA-DR-positive monocytes in circulation was less than 80%. Sixteen healthy volunteers (median age 45 years, range 25 to 66, 13 women) served as reference subjects. The study protocol was approved by the Surgical Ethical Review Board of the Joint Authority for the Hospital District of Helsinki and Uusimaa, and informed consent was obtained from each patient, or their next of kin.

**Table 1:** Characteristics of the patients

	(*n *= 16)
Character	Median (range)
Age, years	47 (30 to 74)
ICU stay, days	23 (4 to 54)
Respirator, days	18 (0 to 42)
Dialysis, days	9 (0 to 40)
Sampling time^1^, days	13 (3 to 39)
CRP at transfer to ICU, mg/l	230 (3 to 566)
APACHE at transfer to ICU	14 (8 to 20)
SOFA at transfer to ICU	5 (4 to 13)
CRP^2^, mg/ml	215 (80 to 451)
APACHE II^2^	15 (6 to 24)
SOFA^2^	8 (2 to 17)
Highest CRP (mg/l)	409 (207 to 566)

APACHE II, Acute Physiology and Chronic Health Evaluation II; CRP, C-reactive protein; SOFA, Sequential Organ Failure Assessment. ^1^Denotes time period from hospital admission to day of blood sampling for lymphocyte functional studies. ^2^At day of blood sampling for lymphocyte functional studies.

The diagnosis of AP was based on typical clinical findings (acute onset of epigastric pain, nausea, and vomiting), elevated serum amylase levels (more than three times the upper reference limit), and/or typical findings in computed tomography performed on all patients within zero to two days after admission to ICU. The AP severity was determined according to the Atlanta classification [24].

### Blood samples and study design

Blood samples were collected within three to five days after the initial determination of the patients' monocyte HLA-DR expression status. Each patient had a reference subject of his own, with the exception of two patients who shared one reference subject. Of the two data sets from this reference subject, the latter was excluded from the data analyses. Parallel peripheral blood samples were obtained from the patient and from his reference subject on two consecutive days, with the exception of one patient, who had different reference subjects on Days 1 and 2. On Day 1, a 4-ml sample of peripheral venous blood was taken (i) for the study of phosphorylation promoted by soluble leukocyte agonists and (ii) for the re-study of the proportion of HLA-DR-positive monocytes. On Day 2, a 4-ml blood sample was taken for the study of leukocyte phosphorylation in response to bacterial cells. The blood samples were collected into Falcon polypropylene tubes (Becton Dickinson, Lincoln Park, NJ, USA) containing pyrogen-free citrate phosphate dextrose (ACD, Baxter Health Care Ltd, Norfolk, England, UK, 0.14 ml/ml blood), kept at the room temperature, and transported within 15 minutes to the laboratory. All aliquots of the blood samples were stimulated within four hours of sampling.

While the study was in progress the patients' lymphocytes showed constitutive STAT3 activation, that is, STAT3 RFU-values in resting cells were consistently higher in patients than healthy subjects. The original protocol was adapted to measure constitutive STAT3 activation and determinate the involved lymphocyte subsets. The revised protocol was applied to patients 11 to 13 and the STAT3 part of the revised protocol to patients 14 to 16 (Table 1).

### Biological agents and leukocyte agonists

We purchased fluorescein isothiocyanate (FITC)-conjugated monoclonal antibodies (mAbs) to CD14 clone MϕP9 (IgG_2b_), CD19 clone SJ25C1 (IgG_1_), CD4 clone SK3 (IgG_1_), and CD8 clone SK1 (IgG_1_), and peridinine-chlorophyll protein (PerCP)- conjugated mAb to CD3 clone SK7 (IgG_1_), phycoerythrin (PE)-conjugated anti-HLA-DR mAb clone L243 (IgG_2a_) and its isotype control (mouse IgG_2a_), Alexa 647-labeled phospho-specific mAbs to pNF-kB p65 (pS529) clone K10-895.12.50 (IgG_2b_), STAT1 (pY701) clone 4a (IgG_2a_), ERK1/2 (pT202/pY204) clone 20A (IgG_1_), and PE-labeled phospho-specific mAbs to STAT3 (pY705) clone 4/P-STAT3 (IgG_2a_) and STAT6 (Y641) clone18 (IgG_2a_) from Becton-Dickinson Biosciences (San Jose, CA, USA). Infliximab (Remicade^®^) was from Schering-Plough Co. (Kenilworth, NJ, USA) and anakinra (Kineret^®^) from Amgen Inc. (Thousand Oaks, CA, USA).

The recombinant cytokines TNF, IL-4, and IL-6 were purchased from R&D (Minneapolis, MN, USA) and phorbol-12-myristate-13-acetate (PMA), *E. coli *O111:B4 lipopolysacharide (LPS), N-acetylmuramyll-alanyl-D-isoglutamine (MDP), and calcium ionophore A23187 from Sigma (St. Louis, MO, USA).

*S. aureus *(IHT 61972) and *E. coli *(IH 3080) were kind gifts from Jaana Vuopio, MD, PhD (The National Institute for Health and Welfare, Helsinki, Finland). *S. epidermidis *was the strain ATCC 53103. Each strain was initially grown in a brain heart infusion (BHI) medium for 18 hours at room temperature (*E. coli*) or at 37°C (*S. aureus and S. epidermidis*). After incubation, the bacteria were pelleted by centrifugation, re-suspended in fresh BHI medium, and further cultured at 37°C for five hours. The secondary cultures were pelleted and washed twice with phosphate buffered saline (PBS). From an aliquot of each cell suspension a diluted culture was made to quantify viable bacteria. The rest of the bacteria were pelleted and re-suspended in a glycerol-tryptone soya broth medium and stored in 1.8-ml aliquots at -70°C.

### Ex vivo stimulation and immunolabeling of blood samples for three-color flow cytometry

Flow cytometry was first used by Fleisher and co-workers [25] to demonstrate intracellular phosphorylation of STAT1 in density gradient-separated monocytes activated *ex vivo *with IFN-γ. We recently developed a whole blood modification of the method [26]. In the present setup, lymphocytes were first delineated according to their light scattering properties using electronic gates and monocytes were recognized by CD14-FITC label positivity and excluded. To study lymphocyte subpopulations, the CD19-FITC-label was used to identify B -lymphocytes and the CD3-PerCP-label was used in combination with the CD4-FITC- or CD8-FITC-labels to identify CD3+CD4+T lymphocytes and CD3+CD8+T lymphocytes. The Alexa647-label was used to detect phosphorylated forms of NF-κB, STAT1, and ERK1/2 and the PE-label to detect phosphorylated STAT3 and STAT6.

The 4-ml blood sample was divided into 90 μl aliquots in Falcon polystyrene tubes (Becton Dickinson, Lincoln Park, NJ, USA) and placed at 37°C. Aliquots of FITC-conjugated mAbs to CD14, CD4, and CD8 were added to the tubes. Next the tubes were supplemented with TNF at the final concentration of 10 ng/ml (10 patients) and incubated for 5 minutes at 37°C, IL-6 100 ng/ml (13 patients) for 5 minutes, IL-4 100 ng/ml (10 patients) for 5 minutes, a combination of PMA 1 μM and calcium ionophore A23187 1 μM (10 patients) for 5 minutes, LPS 100 ng/ml (10 patients) for 10 minutes, MDP 100 ng/ml (10 patients) for 20 minutes, *E. coli *50 cells/leukocyte (10 patients) for 10 minutes, *S. aureus *50 cells/leukocyte (9 patients) for 20 minutes, or *S. epidermidis *50 cells/leukocyte (9 patients) for 40 minutes. Reference tubes were left without stimulus. In one experimental series infliximab (final concentration 10 μg/ml), anakinra (100 μg/ml), or both were mixed with the blood obtained from healthy volunteers before addition of bacteria.

After incubation, red cell lysis, leukocyte fixation, and leukocyte permeabilization were performed according to BD Phosflow Protocol III for Human Whole Blood [27], as described in detail. Briefly, a 1× BD PhosFlow Lyse/Fix Buffer (1.9 ml) pre-warmed to 37°C was added to each tube, they were then incubated for 10 minutes at 37°C, and washed once with Hank's balanced salt solution (Life Technologies, Paisley, UK). For permeabilization, the cell pellet was re-suspended in 1 ml of BD Phosflow Perm Buffer III, pre-cooled at -20°C. The tubes were stored at -20°C until staining with phosphospecific mAbs. After permeabilization, the cell samples were washed twice with ice-cold BD Pharmingen Stain Buffer (BD Sciences, San Jose, CA, USA) and re-suspended in 100 μl of the buffer. Aliquots of Alexa647- and PE-labelled phosphospecific mAbs were then added to the stimulus-treated samples and respective reference samples. Also, aliquots of CD3-PerCP mAb were added at this stage, because PerCP did not withstand the permeabilization procedure. The samples were further incubated in the dark for 30 minutes at 0°C, washed once, and re-suspended in 500 μl of the ice-cold stain buffer. The samples were stored at 0°C and analyzed by flow cytometry within three hours.

Data acquisition and analysis were done with a FACS Calibur flow cytometer and Cell Quest software (BD Sciences, San Jose, CA, USA). A total of 1×10^4 ^lymphocytes, 2×10^3 ^CD3+CD4+ T lymphocytes, 2×10^3 ^CD3+CD8+ T lymphocytes, and 2×10^3 ^CD19+B lymphocytes were collected. Finally, appropriate Alexa647- and PE-histograms were developed to determine the intracellular signaling profiles of the cells.

First, data are presented as median fluorescence intensity, in other words, median RFU (relative fluorescence units) of the whole lymphocyte population, and second, as a proportion of positively fluorescing cells and third, as mean fluorescence intensity of the positively fluorescing cells. If a patient's lymphocytes respond normally, but in reduced number compared to healthy subjects, the mean fluorescence intensity of positively fluorescing cells would remain the same. On the other hand, if a patients' lymphocytes were in a reduced phosphorylation state, this would lead to decreased mean fluorescence intensity of positively fluorescing cells. The proportion of positively fluorescing cells was determined using a threshold method, where an electronic gate was manually set to include the brightest 2 to 4% of the cells in non-stimulated sample. Then the same gate served to determine the proportion of positively fluorescing cells in the respective stimulus-treated sample. Thus, the values < 5% indicate cells not responding to the stimulus. During the study, it emerged that in a proportion of a patient's lymphocyte STAT3 was constitutively active. To determine the proportion of lymphocytes showing constitutive activated STAT3 lymphocytes, the electronic gate of non-stimulated lymphocytes of the healthy subject studied concomitantly was used in the analysis of the patients' samples.

The coefficient of variation was 5% within experiments and 10% between experiments.

The activation of signaling proteins have been confirmed using Western blot analyses by us [26] concerning STAT1 and by Grammer [28] concerning multiple components of NFκB, STAT and MAP-kinase pathways.

Monocyte surface expression of HLA-DR, expressed as the proportion (%) of monocytes positive for HLA-DR fluorescence, was determined as described previously [10].

### Statistical analysis

Results are shown in mean ± SEM or median (range). Using the nonparametric Wilcoxon-Mann-Whitney test we carried out statistical comparisons between the groups. Statistical analyses were performed with SPSS software (v15, SPSS Inc, Chicago, IL, USA). Probabilities were regarded as statistically significant at the 0.05 level.

## Results

### Patients

The etiology for AP was alcohol consumption in 15 patients and biliary stones in 1 patient. Patient characteristics are presented in Table 1. All patients were men. One patient had recurrent AP. All patients developed organ failure: 15 needed mechanical ventilation and 9 needed hemodialysis. Ten patients underwent surgery, 11 had infections, and 3 died due to multiple organ failure (Table 2). The proportion of HLA-DR-positive monocytes was 55.0 ± 4.1% and 93.1 ± 3.4% (*P *< 0.001) in patients and healthy reference subject.

**Table 2:** Outcome of the patients

	(*n *= 16)
Complication	Number of patients
Local complication, number of patients	8
Pancreatic necrosis	5
Pseudocyst	2
Both	1
Operation, number of patients	10
Laparotomy and open abdomen	4
Necrosectomy	5
ERCP	1
Infectious complication	11
Sepsis	2
Abdominal abscess	3
Pneumonia	2
Sepsis and abdominal abscess	2
Abdominal abscess and pneumonia	2
Died^1^	3

ERCP, Endoscopic Retrograde Cholangiopancreatography. ^1^Patients died on the 4^th^, 19^th ^and 24^th ^ICU day.

### NFκB and p38 phosphorylation

**Figure 1:**
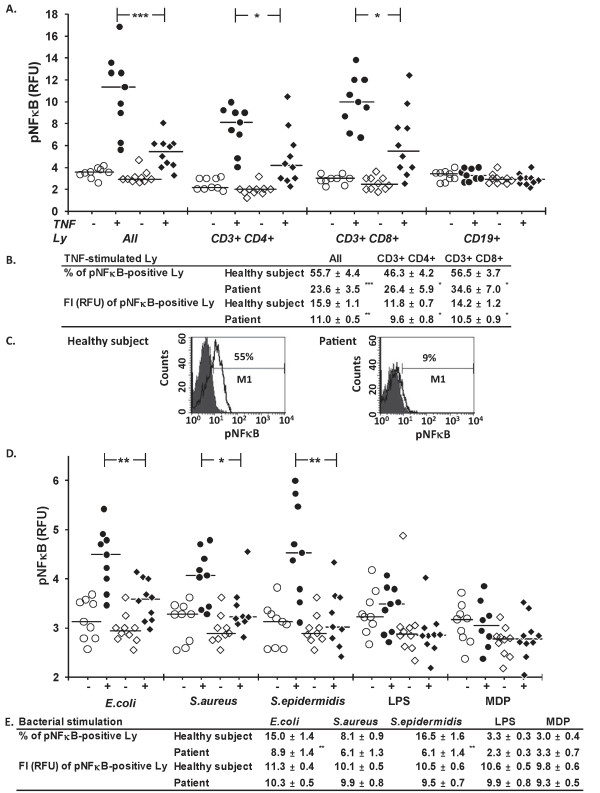
**NF-κB signaling**. Levels of phosphorylated NF-κB p65 (pNF-κB) in lymphocytes (Ly) of healthy subjects (circles) and patients (quadrangles) with acute pancreatitis were measured in whole blood samples left without supplement (open symbols) or supplemented (closed symbols) with TNF (10 ng/ml, five minutes), *E. coli*, *S. aureus*, *S. epidermidis, LPS, or MDP*. Responses to TNF, determined as: **A**. Fluorescence intensity (FI) of all Ly and subsets of Ly (*N *= 9 to 10); **B**. As a proportion of pNFκB-positive Ly and their FI. **C**. The sample histograms of TNF-stimulated (white) and non-stimulated (gray) Ly. The M1 (marker) denotes proportion of pNF-κB-positive Ly. **D**. Responses to bacteria, LPS, and MDP, determined as the FI of all Ly (*N *= 8 to 10). **E**. As proportion of pNFκB-positive cells among all Ly and their FI. RFU, relative fluorescence units. In A and D, horizontal lines denote median, and in B and E, data are given as mean ± SEM. *P < 0.05, **P < 0.01, *** *P *< 0.001.

We used three-color flow cytometry to measure phosphorylation levels of NFκB p65 (pNFκB, Figure 1) and p38 (pp38, Table 3) of lymphocytes in whole blood samples supplemented with TNF or whole bacteria of *E. coli*, *S. aureus*, and *S. epidermidis*. We measured phosphorylation levels in all lymphocytes and, in the case of TNF also in the lymphocytes subclasses, including CD3+CD4+ T lymphocytes, CD3+CD8+ T lymphocytes, and CD19+ B lymphocytes.

**Table 3:** p38 signaling

Stimulation		TNF	LPS	MDP
FI (RFU) of pp38 Ly	Healthy subject	2.5 ± 0.1	1.9 ± 0.1	1.9 ± 0.1
	Patient	3.4 ± 0.5*	2.1 ± 0.1	2.0 ± 0.1
% of pp38 positive Ly	Healthy subject	18.4 ± 2.3	3.4 ± 0.3	3.4 ± 0.4
	Patient	22.1 ± 2.9	4.8 ± 0.6	3.2 ± 0.3
FI (RFU) of pp38 positive Ly	Healthy subject	6.4 ± 0.3	5.5 ± 0.4	5.1 ± 0.4
	Patient	7.1 ± 0.6	6.6 ± 1.2	7.3 ± 1.8
				
**Stimulation**		***E. coli***	***S. aureus***	***S. epidermidis***
FI (RFU) of pp38 Ly	Healthy subject	2.0 ± 0.1	2.1 ± 0.1	2.1 ± 0.1
	Patient	2.7 ± 0.2**	2.9 ± 0.3*	2.2 ± 0.1
% of pp38 positive Ly	Healthy subject	8.3 ± 0.9	6.1 ± 0.6	6.9 ± 0.7
	Patient	15.6 ± 1.8**	16.0 ± 3.5*	5.4 ± 0.9
FI (RFU) of pp38 positive Ly	Healthy subject	6.3 ± 1.0	5.2 ± 0.4	5.1 ± 0.3
	Patient	6.9 ± 0.7	7.5 ± 1.1	7.3 ± 1.3

FI, fluorescence intensity; LPS, lipopolysaccharide; Ly, lymphocytes; MDP, N-acetylmuralyl-alanyl-D-isoglutamine; TNF-α, tumor necrosis factor-α. *N *= 9 to 10, *P < 0.05, **P < 0.01 as compared to healthy subject

Figure 1A shows that TNF stimulation increased pNFκB fluorescence intensity values in all lymphocytes, and in CD3+CD4+ and CD3+CD8+ lymphocyte subsets, but not in CD19+ lymphocyte subset of healthy subjects. In patients, the TNF-induced responses of all lymphocytes, CD3+CD4+ lymphocytes, and CD3+CD8+ lymphocytes were significantly lower than those of healthy subjects. The pNFκB levels of the non-stimulated lymphocytes were comparable.

Further analysis of the data (Figure 1B) showed that the proportions of pNFκB-positive lymphocytes in TNF-treated samples were lower in patients than in healthy subjects among all lymphocytes, CD3+CD4+ T lymphocytes, and CD3+CD8+ T lymphocytes, indicating that patients had a reduced number of TNF responding cells. In addition, the pNFκB fluorescence intensity of the TNF responding cells was lower in patients than in healthy subjects, in all lymphocytes, CD3+CD4+ cells and CD3+CD8+ cells. The shape of lymphocyte histograms (Figure 1C), and those of lymphocyte subsets (not shown), were uniform and did not reveal any lymphocyte subpopulations.

Whole bacteria, but not LPS or MDP, increased the pNFκB levels of all lymphocytes in blood samples from healthy subjects (Figure 1D). The pNFκB fluorescence intensities induced by whole bacteria were lower in patients than in healthy subjects. The proportions of pNFκB-positive lymphocytes were significantly lower in patients' cells than control cells exposed to *E. coli *and *S. epidermidis *(Figure 1E), indicating that patients had fewer responding cells. The difference in pNFκB fluorescence intensities of the responding cells between patients and healthy subjects was not significant.

**Figure 2:**
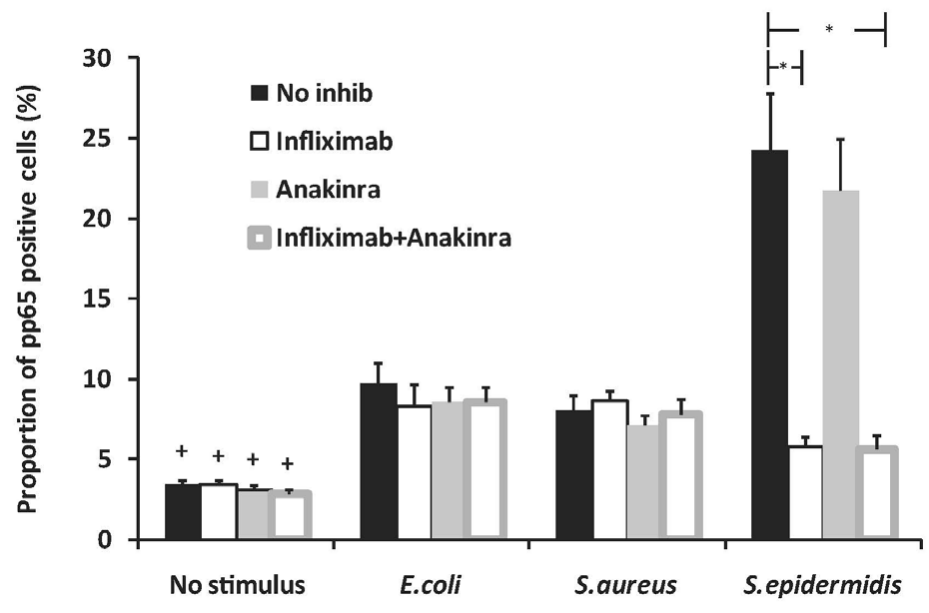
**The effects of infliximab and anakinra on bacteria-induced NF-κB phosphorylation in lymphocytes**. Whole blood samples of healthy subjects were left without cytokine inhibitor or mixed with infliximab, an anti-TNF mAb, anakinra, an IL-1 receptor antagonist, or both, and then left without further supplement or supplemented with *E. coli*, *S. aureus*, or *S. epidermidis*. *P < 0.05 (*N *= 4 to 6). ^+ ^Significantly different (*P *< 0.05) from respective *E. coli, S. aureus*, and *S. epidermidis *groups with the exception of *S. epidermidis *with infliximab only (*P *= 0.055), or infliximab + anakinra (*P *= 0.054).

We supplemented the culture tubes with anti-TNF mAb infliximab, IL-1ra anakinra, or a combination of them to investigate whether bacteria-induced lymphocyte activation was secondary to phagocyte-derived cytokines (Figure 2). Infliximab decreased, although not completely, NFκB activation induced by *S. epidermidis*, but not by *E. coli *or *S. aureus*. Anakinra had no effect on pNFκB levels.

The fluorescence intensity of pp38 of all lymphocytes, induced by TNF, *E. coli*, or *S. aureus*, was higher in patients than in healthy subjects (Table 3). The proportions of positively fluorescing cells, induced by *E. coli *or *S. aureus*, were higher in patients. The fluorescence intensity of the pp38-positive cells was also higher in patients than controls, but the difference was not statistically significant.

### ERK1/2 phosphorylation

**Figure 3:**
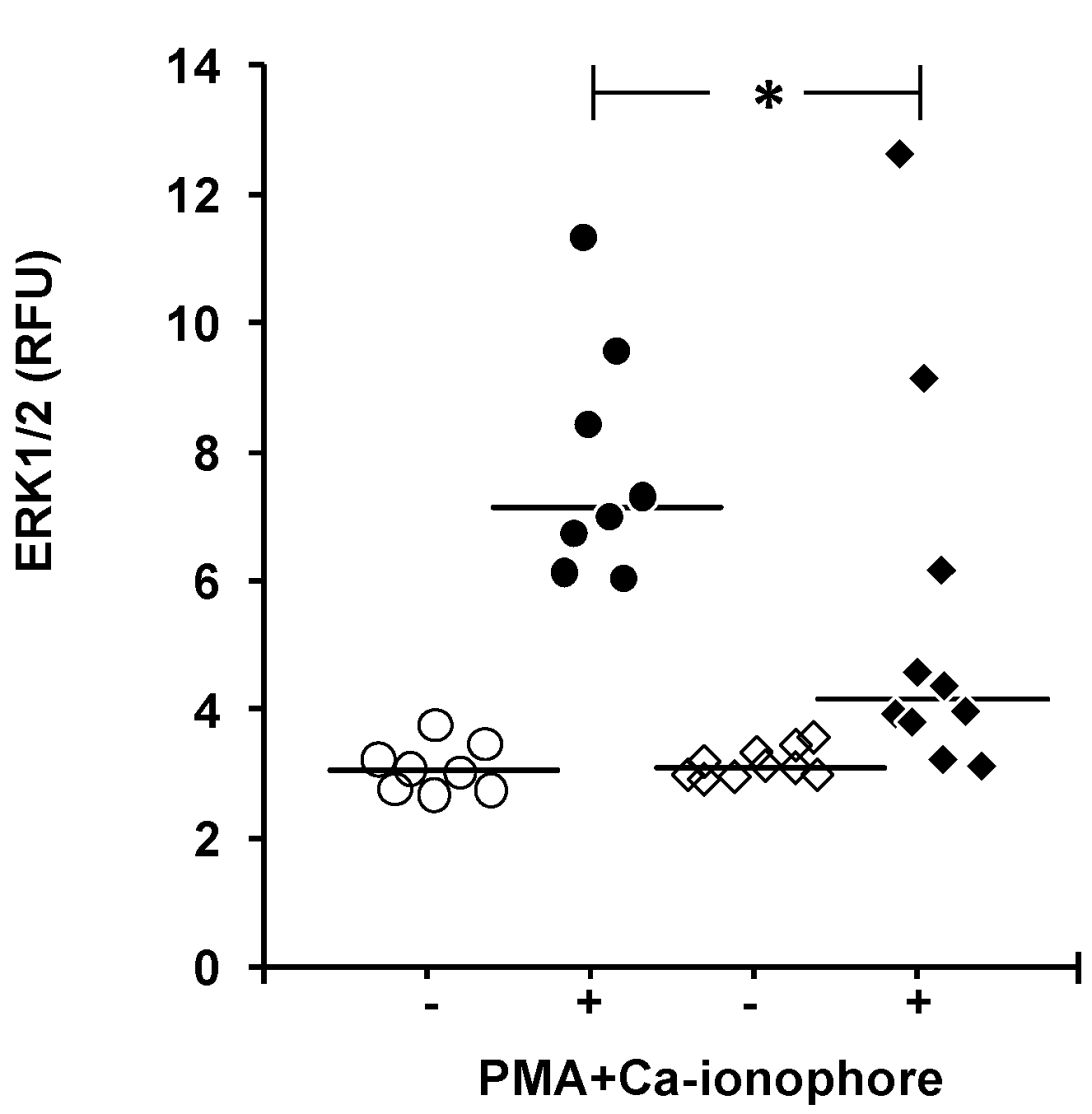
**ERK1/2 signaling**. Levels of ERK1/2 phosphorylation in lymphocytes of healthy subjects (circles) and patients (quadrangles) were measured in whole blood samples without supplement (open circles) or supplemented (closed symbols) with combination of PMA (1 μM) and Ca-ionophore (1 μM). RFU, relative fluorescence units. *P < 0.05 (*N *= 8 to 10).

The pERK1/2 levels of all lymphocytes induced by the combination of PMA and calcium ionophore were lower in the patients (*P *= 0.034, Figure 3). The proportion of pERK1/2-positive lymphocytes was also lower in the patient group (22.8 ± 7.8% vs 43.3 ± 5.1%, *P *= 0.043), indicating that the responding cells in patients were decreased. The fluorescence intensity of pERK1/2 positive cells of patients and healthy subjects were comparable (12.2 ± 0.6 RFU vs. 11.5 ± 0.6 RFU, *P *> 0.05).

### STAT3 phosphorylation

**Figure 4:**
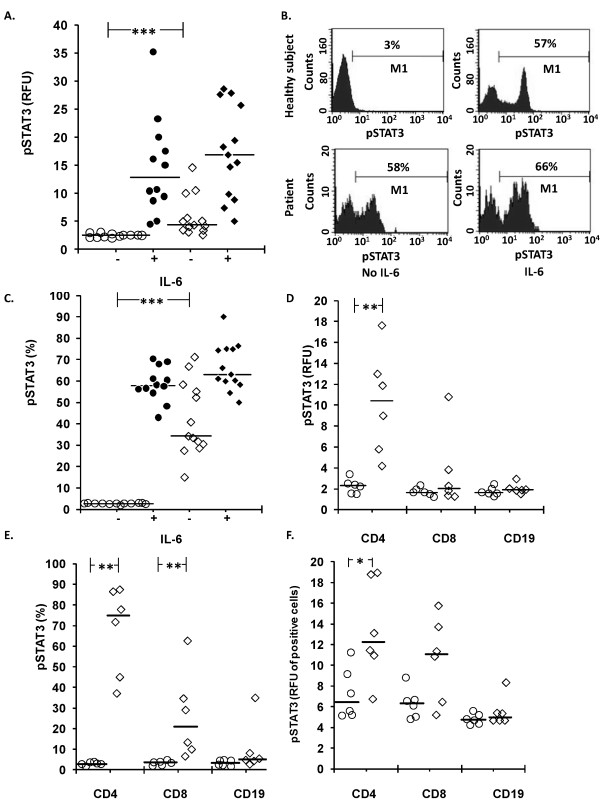
**STAT3 signaling**. Levels of phosphorylated STAT3 (pSTAT3) in lymphocytes of healthy subjects (circles) and patients (quadrangles) in whole blood samples without supplement (open symbols) or supplemented (closed symbols) with IL-6 (100 ng/ml, five minutes). **A**. Fluorescence intensity (RFU, relative fluorescence units); **B**. sample histograms; **C**. proportion of pSTAT3-positive cells among all lymphocytes (*N *= 12 to 13); **D**. Fluorescence intensity; **E**. proportion of pSTAT3-positive lymphocytes among; **F**. fluorescence intensity of pSTAT3-positive lymphocytes among subsets of non-stimulated lymphocytes. Horizontal lines in A and C-F denote median and M1 (marker) in B denotes proportion of pSTAT3-positive lymphocytes. *P < 0.05, **P < 0.01, *** *P *< 0.001.

In the non-stimulated samples, the pSTAT3 fluorescence intensity of all lymphocytes was significantly higher in patients than healthy subjects (*P *< 0.001, Figure 4A). The shapes of the patients' pSTAT3 histograms were biphasic (Figure 4B), indicating the presence of an activated cells subset. To evaluate the proportions of pSTAT3 positive cells in patient samples, the electronic gate of healthy subjects lymphocytes was applied to the patient lymphocytes. The proportion of pSTAT3-positive cells was higher in patients than in healthy subjects (42.0 ± 4.7% vs 2.6 ± 0.1%, *P *< 0.001, Figure 4C).

In the IL-6-treated samples, the proportion of pSTAT3 positive lymphocytes in patients was 66.5 ± 3.1% and in healthy subjects 58.6 ± 2.3% (*P *> 0.05). The fluorescence intensity values of positively fluorescing cells of patients and healthy subjects were 27.1 ± 2.2 and 25.5 ± 2.8 (*P *> 0.05).

The lymphocyte subgroup analysis of non-stimulated cells showed that patients' CD3+CD4+ lymphocytes, compared to reference cells, had higher pSTAT3 levels, defined as fluorescence intensity of all CD3+CD4+ cells (Figure 4D), the proportion of pSTAT3-positive cells (Figure 4E), and fluorescence intensity of pSTAT3-positive cells (Figure 4F). The proportion of pSTAT3 positive CD+CD8+ lymphocytes was higher in patients (Figure 4E).

### STAT1 and STAT6 phosphorylation

**Figure 5:**
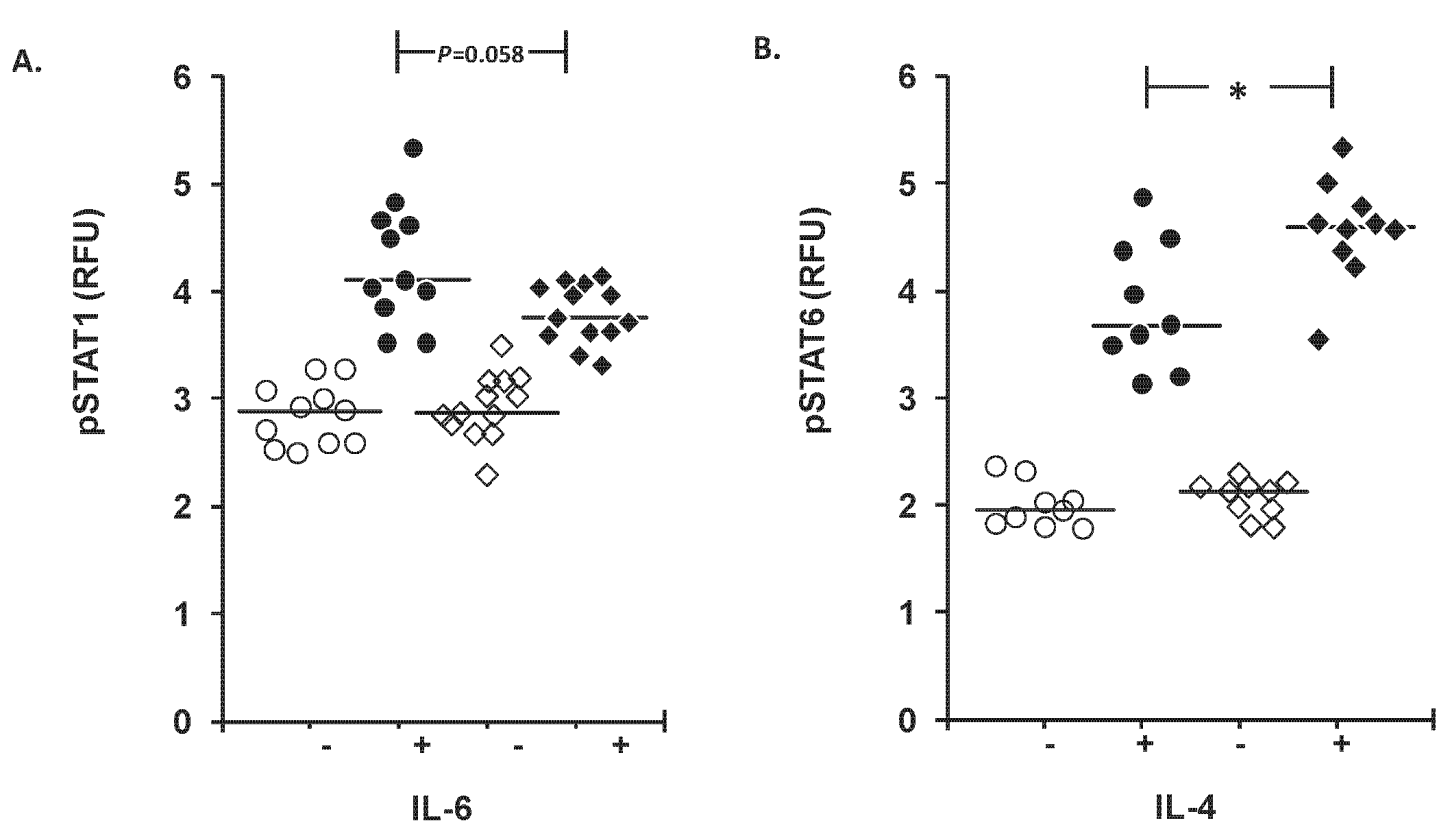
**STAT1 and STAT6 signaling**. Levels of **A**. pSTAT1 fluorescence intensity (FI); **B**. pSTAT6 FI in lymphocytes (Ly) of healthy subjects (circles) and patients (quadrangles) in whole blood samples left without supplement (open symbols) or supplemented (closed symbols) with IL-6 (100 ng/ml, five minures) in A and IL-4 (100 ng/ml, five minutes) in B. RFU, relative fluorescence units. *P < 0.05.

The patients had lower IL-6 induced pSTAT1 levels than the healthy subjects, determined as fluorescence intensity of all lymphocytes (*P *= 0.058, Figure 5A), proportion of pSTAT1 positive cells, and fluorescence intensity of pSTAT1 positive cells (*P *< 0.05, Table 4).

**Table 4:** The proportions of pSTAT1- or pSTAT6-positive lymphocytes (Ly) and their fluorescence intensity (FI)

Transcription factor		pSTAT1	pSTAT6
Stimulation		IL-6	IL-4
% of pSTAT-positive Ly	Healthy subject	22.5 ± 2.8	37.0 ± 5.8
	Patient	13.8 ± 1.6*	47.6 ± 5.1
FI (RFU) of pSTAT-positive Ly	Healthy subject	11.4 ± 0.4	5.8 ± 0.3
	Patient	9.7 ± 0.2*	6.3 ± 0.3

RFU, relative fluorescence units. *P < 0.05

The pSTAT6 levels of all lymphocytes induced by IL-4 were higher in patients than in healthy subjects (Figure 5B). The proportion of pSTAT6 positive cells and the fluorescence intensity of them were also higher in patients, but the difference was not significant (Table 4).

## Discussion

Our results show multiple aberrations in pro- and anti-inflammatory signaling pathways of lymphocytes, determined by phospho-specific whole blood flow cytometry, in AP patients with vital organ dysfunction and immune suppression. Patients' lymphocytes showed decreased NFκB phosphorylation in whole blood samples supplemented with exogenous TNF. The defect appeared to involve all TNF-responding lymphocytes rather than a subset, because the pNFκB histograms were uniform. Both the proportion of pNFκB-positive cells and their fluorescence intensity were also reduced among CD3+CD4+ and CD3+CD8+ T lymphocytes. NFκB phosphorylation promoted by *E. coli*, *S. aureus*, and *S. epidermidis *was also reduced. Lymphocyte activation was probably secondary to phlogistic mediators generated in whole blood samples supplemented with bacteria. Indeed, the TNF-blocking agent infliximab in co-culture with *S. epidermidis*, but not with *E. coli *or *S. aureus*, reduced NFκB phosphorylation, while the IL-1 receptor antagonist, anakinra, had no effect, suggesting that lymphocyte activation involved TNF among other factor(s). We have recently found that monocytes of the patients also showed reduced NFκB phosphorylation in response to bacterial stimuli [23], which agrees with our finding that TNF production by anergic monocytes is reduced [29]. Collectively, the above data shows disturbances in collaboration between patients' lymphocytes and monocytes. The impaired collaboration, together with a significant reduction of circulating T- and B-lymphocytes in severe AP [13,30] may contribute to the development of secondary infections in the patients. The risk of infections, however, remains to be determined in prospective follow-up studies of leukocyte NFκB phosphorylation profiles in relation to clinical outcome.

In contrast to NFκB activation, the proportion of pp38-positive lymphocytes induced by *E. coli *or *S. aureus *were higher in patients than reference subjects, indicating that patients had an increased number of responding cells. Of note, the lymphocytes were double-stained with pNFκB and pp38 mAbs, and, consequently, activity of the two signaling pathways could be evaluated simultaneously. Unlike lymphocytes, the p38 phosphorylation of the patients' monocytes was normal [23]. Given that MAP-kinases are associated with the development of systemic inflammation [14], our finding raises the question of whether enhanced p38 activation provides a target for immune suppression in AP patients or if it represents a vital counter-reaction of cells to inhibit NFκB, and should therefore be strengthened rather than depressed.

Phosphorylation of the other MAP-kinase protein ERK1/2 was depressed in this study. ERK1/2 is functionally related to the migration of inflammatory cells, including lymphocytes [31]. Monocytes of our patients showed low pERK1/2 levels and poor transmigration *in vitro*, whereas their pp38 levels were normal [23]. The possibility that the inhibition of p38 expression and upregulation of ERK1/2 expression is beneficial in human AP, as suggested by studies in experimental AP [32], warrants further studies.

The results show that STAT3 is constitutively activated in the patients' lymphocytes, particularly in CD3+CD4+ and CD3+CD8+ T-lymphocytes. The constitutive STAT3 activation was confined to lymphocytes and did not occur in the patients' monocytes [23]. Constitutive STAT3 expression has been described in a variety of disorders [33-35] and may involve a complex crosstalk between different signaling pathways [36]. Because STAT3 mediates anti-inflammatory signals its constitutive activation may denote an attempt to down-regulate inflammation. The activation was partial and could be completed with IL-6, which promoted pSTAT3 levels in patients' lymphocytes similar to those in reference lymphocytes of healthy subjects. Unlike lymphocytes, the IL-6 induced STAT3 phosphorylation of the patients' monocytes was depressed [23]. Thus, STAT3 signaling pathway was more impaired in monocytes than in lymphocytes. In addition to IL-6, STAT3 is activated by IL-10, and circulating levels of both are elevated in AP patients [11], thus possibly contributing to the constitutive pSTAT3 expression. In this context it is of interest that IL-6 together with IL-21 and IL-23 promote sustained STAT3 activation, which favors the Th17 developmental program of CD4+ lymphocytes [18]. Serum levels of IL-17 were also elevated and served as prognostic markers in patients with severe AP [37]. The Th17 immune pathway has not, to our knowledge, been thoroughly explored in patients with AP. If involved in the pathogenesis of tissue injury in severe AP, the Th17 pathway may reveal novel prognostic markers and therapeutic possibilities.

STAT1 is activated by multiple cytokines [15,16] and associated with pro-inflammatory signaling and development of inflammatory tissue injury. We used IL-6 to phosphorylate STAT1 and found lower levels in patients' lymphocytes than in reference cells. The proportion of pSTAT1-positive cells and their fluorescence intensity were also lower, indicating that the defect involved all IL-6-responding lymphocytes rather than a subset of them. Also the patients' monocytes were depressed [23]. Unlike pSTAT1 levels, the IL-4-induced levels of p-STAT6, which mediates Th2 signals and is associated with less injurious tissue reactions, were higher in patients' cells. The results suggest that the patients' immune system tries to inhibit inflammatory tissue injury by shifting the tissue-destructive Th1 type of immune response to the less injurious Th2 type. Our results are in accordance with the findings regarding experimental AP [12] and patients with AP, indicating that although both Th1 and Th2 cytokines are elevated, the magnitude of elevation of the latter is much higher [13].

Taken together, our data concerning the patients' lymphocytes and monocytes indicate that signaling pathways are more impaired in monocytes. The strong depression of monocytes is meaningful because monocytes are powerful mediators of tissue destruction.

Despite our findings being in accordance with clinical findings and current concepts of immune pathogenesis of severe systemic inflammation, the data should be interpreted with caution. First, the study was confined to a limited number of patients, who had vital organ dysfunction and whose clinical outcome and immune inflammatory status were reasonably comparable. We focused on these patients to screen for the affected signaling profiles in the most severe form of AP. In the future, the aberrant profiles of lymphocytes and monocytes [23] need to be studied prospectively during follow-up of patients with varying AP severity. Second, phospho-specific whole blood flow cytometry is susceptible to methodological errors. A meticulous sample handling is needed to avoid inappropriate cell activation *ex vivo *[23]. Another critical step is permeabilization of the cells, because it permits the phospho-specific antibody molecules to enter the intracellular compartments. Leukocyte activation may increase cellular resistance to membrane-active agents [38]. Although we cannot exclude the possibility that patient lymphocytes are more resistant to permeabilization, such a difference is not supported by our findings that pSTAT3 levels were constitutively increased in the patient lymphocytes and double-stained lymphocytes showed simultaneously enhanced p38 activation and depressed NFκB activation in the same cell. Considering caveats, our present results and previous studies [21,23,26] suggest that whole blood phosphor-specific flow cytometry is a suitable method for immune monitoring of patients with systemic inflammation.

## Conclusions

Our results show a variety of aberrations in the signaling profile of lymphocytes, which are in accordance with clinical data and the immune status of the patients. Although the data are preliminary, because of confinement to a limited number of patients with the most severe disease form, the results encourage study of the possibility that prospective monitoring of lymphocyte signaling profiles may aid in predicting AP outcome and provide novel targets for immune therapy.

## Key messages

• Signaling profiles of lymphocytes provide a novel means for immune monitoring of patients with systemic inflammation.

• Our data show, for the first time, that in acute pancreatitis complicated by vital organ dysfunction multiple aberrations occur in lymphocyte signaling profiles.

• The possibilities that the aberrations predict organ dysfunction and reveal novel means for targeted therapy warrant further studies.

## Abbreviations

AP: acute pancreatitis; APACHE II: Acute Physiology and Chronic Health Evaluation II; BHI: brain heart infusion; CRP: C-reactive protein; E. coli: Escherichia coli; ERCP: Endoscopic Retrograde Cholangiopancreatography; ERK: extracellular signal regulated kinase; FI: fluorescence intensity; FITC: fluorescein isothiocyanate; HLA-DR: human leukocyte antigen -DR; IFN-γ: interferon-γ; IL: interleukin; IL-1ra: IL-1 receptor antagonist; LPS: lipopolysaccharide; Ly: lymphocyte; MAP: mitogen activated protein; MDP: N-acetylmuralyl-alanyl-D-isoglutamine; NFκB: nuclear factor κB; pNFκB: phosphorylated NFκB; PBS: phosphate buffered saline; PE: phycoerythrin; PerCP: peritidine-chlorophyll protein; PMA: phorbol-12-myristate-13-acetate; pp38: phosphorylated p38; pSTAT: phosphorylated STAT; RFU: relative fluorescence unit; S. aureus: Staphylococcus aureus; S. epidermidis: Staphylococcus epidermidis; SEM: standard error of the mean; SOFA: Sequential Organ Failure Assessment; STAT: signal transducer and activator of transcription; TNF-α: tumor necrosis factor-α

## Competing interests

The authors declare that they have no competing interests.

## Authors' contributions

JO collected clinical data and participated in data analyses and the writing of the manuscript. HM participated in design of the study and drafting of the manuscript and performed statistical analysis. MLK and LK participated in design and coordination of the study and drafting of the manuscript. KK participated in flow cytometry and drafting of the manuscript. SS was responsible for the flow cytometry and participated in drafting of the manuscript. EK and PP participated in design and coordination of the study and helped draft the manuscript. HR conceived the study, participated in its design, and helped draft the manuscript.
